# Local Dot Motion, Not Global Configuration, Determines Dogs’ Preference for Point-Light Displays

**DOI:** 10.3390/ani9090661

**Published:** 2019-09-06

**Authors:** Carla J. Eatherington, Lieta Marinelli, Miina Lõoke, Luca Battaglini, Paolo Mongillo

**Affiliations:** 1 Laboratory of Applied Ethology, Department of Comparative Biomedicine and Food Science, University of Padua, Viale dell’Università 16, 35020 Legnaro, Italy (C.J.E.) (M.L.) (P.M.); 2 Department of General Psychology, University of Padua, Via Venezia 8, 35131 Padova, Italy

**Keywords:** dog, biological motion, point-light display, visual perception, experience

## Abstract

**Simple Summary:**

Animal motion is characterised by predictable kinematics according to their body morphology and the laws of gravity. This pattern of movement, called biological motion, is traditionally studied using animated displays created by placing a small number of light dots on the major joints of living beings. Previous studies have shown that several animal species can reliably discriminate dot displays depicting an animal walking, and their performance is impeded when the display is turned upside-down and is variably affected when each dot is displaced to disrupt the global biological arrangement. In this study, we investigated this phenomenon in dogs during the presentation of dot displays depicting humans or dogs walking. Our findings showed that dogs preferred to view the display which depicted an upright dog, regardless of its global arrangement, and had no significant preferences when displays depicting humans were presented. This suggests that dogs’ sensitivity to biological motion depends mainly on the presence of dot motion that moves in accordance with gravity. Also, our findings suggest that, despite dogs’ extensive exposure to human motion, they are not sensitive to the bipedal motion presented in the human dot displays.

**Abstract:**

Visual perception remains an understudied area of dog cognition, particularly the perception of biological motion where the small amount of previous research has created an unclear impression regarding dogs’ visual preference towards different types of point-light displays. To date, no thorough investigation has been conducted regarding which aspects of the motion contained in point-light displays attract dogs. To test this, pet dogs (*N* = 48) were presented with pairs of point-light displays with systematic manipulation of motion features (i.e., upright or inverted orientation, coherent or scrambled configuration, human or dog species). Results revealed a significant effect of inversion, with dogs directing significantly longer looking time towards upright than inverted dog point-light displays; no effect was found for scrambling or the scrambling-inversion interaction. No looking time bias was found when dogs were presented with human point-light displays, regardless of their orientation or configuration. The results of the current study imply that dogs’ visual preference is driven by the motion of individual dots in accordance with gravity, rather than the point-light display’s global arrangement, regardless their long exposure to human motion.

## 1. Introduction

Animal motion is characterised by predictable kinematics according to their body morphology and the laws of gravity. Johansson [1] captured this movement by placing a small number of point-lights on the major joints of a human body and found that when viewed in isolation they still created the impression of a moving person—despite the lack of other visual information. Biological motion perception has been extensively researched in humans, the results of which demonstrate that people are able to extract a wealth of information from point-light displays, including gender [2,3], emotional state [4], familiarity [5,6] and action performed [7,8]. The perception of biological motion is also relevant to non-human animals, although instead of assessing their ability to infer specific information from point-light displays, research has tended to focus on demonstrating the relevance of biological motion cues to the species under investigation via conditioned discrimination (Baboons [9]; Bottlenose dolphins [10]; Cats [11]; Chimpanzees [12]; Pigeons [13,14]; Rats [15]) or spontaneous preference tasks (e.g., Dogs [16,17]; Chicks [18,19,20,21]; Medaka fish [22]; Marmosets [23]; Mice [24]).

In order to assess an individual’s preference towards biological or non-biological motion, point-light displays are often presented in conjunction with manipulated ones. A common stimulus manipulation is to flip the point-light display along the horizontal axis. Inverting a point-light display preserves the spatial relationship between dots, but alters the movement of individual dots, which no longer conform to the laws of gravity. Studies conducted in human infants [25] and visually naive chicks [20] have revealed that this disruption of local dot motion reduces the attractiveness of point-light displays, so they are viewed for less time. It has also been shown that inverting a point-light display impaired cats’ [11], marmosets’ [23] and pigeons’ [14] ability to discriminate biological motion. This influence is particularly apparent in dots representing wrists or ankles, which are therefore believed to represent crucial cues for detecting biological motion [26].

A different common manipulation is to scramble point-light displays, by moving individual dots to a different starting position. Scrambling a point-light display disrupts the spatial relationship between dots but maintains the trajectory and accordance with gravity of local dot motions. The impact of disrupting the display’s global structure whilst preserving the local dots motion is less clear and may be dependent on the species under investigation. For instance, research in human infants [25] and chicks [19] revealed no visual preference towards coherent or scrambled point-light displays. On the other hand, mice [24], female marmosets [23] and female chicks [18] looked significantly less towards scrambled point-light displays than coherent displays. Blake [11] also found that scrambling a point-light display impaired cats’ ability to discriminate biological motion. However, Parron and co-authors [9] found a higher rate of discrimination transfer between upright coherent point-light displays to scrambled point-light displays than from upright coherent point-light displays to inverted coherent point-light displays. Also, the finding that the inversion effect can still be detected when displays are scrambled [26] suggests that the global structure of a point-light display may be less important for biological motion perception than the motion of individual dots in accordance with gravity.

To date, limited investigation into biological motion perception in dogs has been conducted. The first to present animated point-light displays to dogs were Kovács and co-authors [16], who investigated the role of oxytocin on dogs’ sensitivity to human motion. In their study, pairs of stimuli were presented comprising an upright coherent human and an inverted and scrambled human, with or without a background of random dots. The experiment revealed a significant effect of oxytocin in modulating dogs’ looking preference, when the point-light displays were not masked by random dots, implying that reducing dogs’ responsivity influenced their preference for different types of point-light displays. However, no direct comparison of dogs’ visual preference to either stimulus was presented, thus leaving unanswered the question of whether dogs do show a visual preference bias towards human biological motion.

Ishikawa and co-authors [17] investigated the role of sociability on dogs’ preference for viewing conspecific and human point-light displays. Several combinations of stimuli pairs were presented, varying in terms of manipulation (upright or inverted orientation), direction of movement (frontal or lateral) and species (dog or human). Dogs’ level of sociability towards humans and dogs was measured via a questionnaire completed by the owner, which allowed researchers to categorise dogs into high or low sociability groups. A complex pattern of results revealed that overall dogs looked significantly more at human upright frontal point-light displays compared to their inverted control. However, they also found that high-sociability dogs preferentially viewed human inverted point-light displays when presented in the lateral orientation compared to its upright counterpart. And finally, that although low sociability dogs preferentially orientated towards upright dog displays presented laterally compared with its frontally orientated control, high-sociability dogs exhibited the diametrically opposite pattern of results.

In summary, the two previous studies into biological motion perception in dogs [16,17] were not able to clearly answer to what types of point-light displays dogs are preferentially attracted. A possible reason for this is because different types of point-light displays contain one or more different motion features (e.g., upright, inverted, coherent, scrambled). Consequently, the aim of the current experiment was to better understand what features of point-light displays dogs preferentially view, by systematically manipulating physical aspects of point-light displays representing both dogs’ and humans’ motion.

## 2. Materials and Methods

### 2.1. Subjects

Forty-eight dog-owner dyads were recruited through the database of volunteers at the Laboratory of Applied Ethology in the University of Padua. Twenty-eight dogs were pure-breeds (4 Australian Shepherds, 4 Border Collies, 3 Cocker Spaniels, 3 German Shepherds, 3 Golden Retrievers, 2 Weimaraners, 1 Basenji, 1 Czechoslovakian Wolfdog, 1 Dogue de Bordeaux, 1 English Setter, 1 Greyhound, 1 Staffordshire Bull Terrier, 1 Standard Poodle, 1 Vizsla, 1 Whippet) and 20 were mixed-breed dogs (4 small, ≤35 cm at the withers; 10 medium, >35 and <55 cm; 6 large, ≥55 cm). The sample consisted of 29 females and 19 males (mean age ± SD: 5.3 ± 2.6 years). The criteria for recruitment were that dogs had lived with their current owner for the last six months and that they were in good health condition. The study was conducted in accordance with relevant legislation about research involving animals, and, for the type of procedures involved, no formal ethical approval was required.

### 2.2. Stimuli

**Figure 1:**
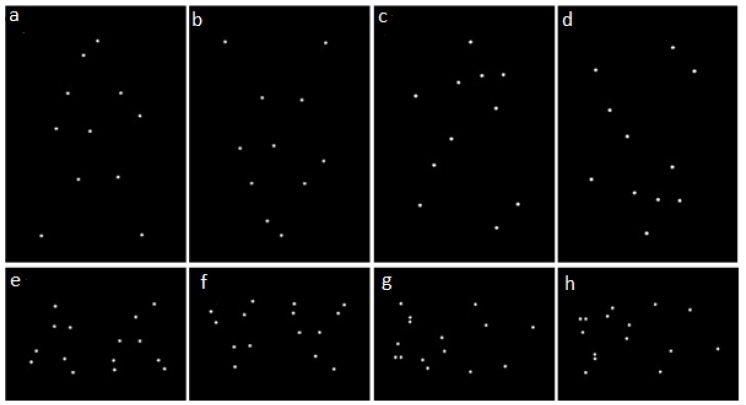
Screenshots exemplifying different types of stimuli used in the experiment: (**a**) human upright coherent, (**b**) human inverted coherent, (**c**) human upright scrambled, (**d**) human inverted scrambled, (**e**) dog upright coherent, (**f**) dog inverted coherent, (**g**) dog upright scrambled and (**h**) dog inverted scrambled.

The stimuli consisted of white point-light displays representing walking humans or dogs on a black background (Figure 1). The point-light displays were created by video recording one male and one female from each species walking with lateral orientation from left-to-right or right-to-left at a constant speed for one complete cycle of their legs. Markers were placed on the following joints: atlas-occipital, shoulder, elbow, wrist, hip, knee, ankle and, for dogs only, the metatarsophalangeal and metacarpophalangeal joints. Videos were recorded at 120 frames per second. The videos were stabilised using Adobe After Effects CC 2017 (Version 14.2.1, Adobe Inc., San Jose, CA, USA), so they looked as if the person/dog was walking on a treadmill. The resultant movie clip was imported into Tracker [27], where the coordinates for each joint marker were recorded frame-by-frame. Using these coordinates, point-light animations were created using the BioMotion Toolbox [28] for Matlab (Mathworks Inc., Natick, MA, USA). The resulting animation was looped to create 15 s presentations with continuous motion. The BioMotion Toolbox was also used to create inverted and scrambled versions of the original point-light displays. In inverted versions the overall point-light displays were flipped upside-down, so that the spatial relationships between individual points were maintained, but the characteristics of their local motion were opposed to that of a biological entity with respect to gravity. In scrambled versions each individual dot composing the point-light display was randomly displaced to a different starting position compared to its original location, thereby disrupting the global coherence of the point-light display, whilst maintaining the characteristics of the local motion with respect to gravity. Inversion and scrambling could be combined to obtain inverted-scrambled point-light displays, so four different types of stimuli were created for both human and dog stimuli: upright coherent (UC), inverted coherent (IC), upright scrambled (US) and inverted scrambled (IS) (see Figure 1). Moreover, two versions of each stimulus were created, one where the animated figure appeared to be facing right and one facing left.

### 2.3. Experimental Setting

**Figure 2:**
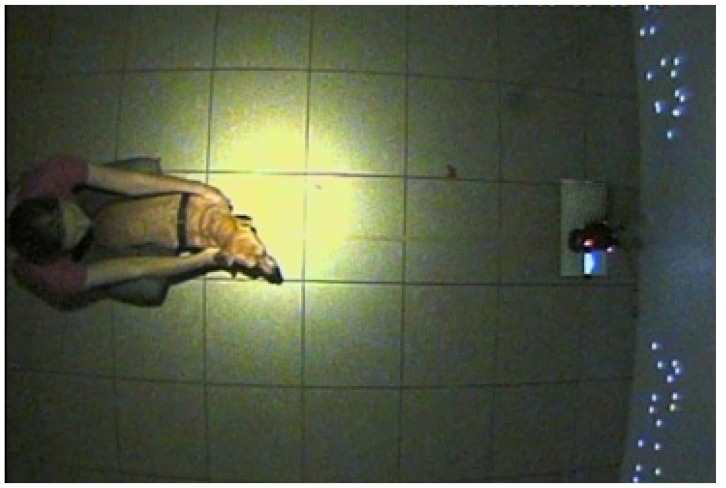
A video-still of the experimental setting, during a presentation.

The experiment was conducted in a quiet, dimly lit (approximately 4 cd/m^2^) room (4.7 × 5.8 m) with a large plastic screen (2.4 × 3.4 m) at one end and a Toshiba TDP T100 projector (Toshiba corporation, Tokyo, Japan) mounted 2.15 m high on the wall opposite. Pairs of stimuli were projected onto the screen simultaneously. Human stimuli were sized approximately 130 × 80 cm and dog stimuli were sized approximately 65 × 100 cm. The distance between the centre of each point-light display was 1.80 m. During testing, dogs faced the screen at a distance of 1.65 m, either standing or sitting in between their owner’s legs who was seated on a small stool behind them (Figure 2). Owners were instructed to gently hold the dog in place but look straight ahead so as not to influence the dog’s behaviour. Trial presentation was controlled by an experimenter seated at the back of the room, using a MacBook Pro. A Canon XA20 (Canon, Tokyo, Japan) camcorder was mounted at floor level, 10 cm in front of the screen and facing the dog’s head, to record dog’s eye movements. Finally, two CCTV cameras were mounted on the ceiling, facing down towards the dog to record its head orientation.

### 2.4. Experimental Design

For each species, all possible combinations of manipulations were paired, therefore obtaining six different trial types per species, that displayed two different stimuli simultaneously (see Table 1). The two stimuli within each trial type were facing the same direction. To contain experimental subject habituation, each dog was only presented with three trial types per species (three dog point-light displays and three human point-light displays), totalling six trials per dog; in addition, the direction of movement of the figures was randomised and counterbalanced among the six trials. Overall, each trial type, for both dog and human point-light displays, was seen by 24 subjects. The presentation order of the six trials was pseudo-randomised, to ensure that each trial type was presented equally as often first, second, third, fourth, fifth or sixth, and that human and dog trials were presented in alternate fashion. Also, the side of presentation of the two stimuli, and the direction of movement of the figures, were counterbalanced across the dogs’ sample.

### 2.5. Test Procedure

Dogs were initially given ten minutes to become familiar with environment, including the experimenter. Before each trial, dogs were led into the testing room and positioned in front of the screen with their two left and two right paws either side of a central line marked on the floor. Each trial was started when dog was looking straight forward towards the presentation screen; and if the dog did not express the behaviour spontaneously, then their attention was captured by moving the projected computer mouse. At the start of the trial, the two point-light displays composing the trial-type were projected, and held on for 15 s, after which the stimuli disappeared, and a black screen appeared. Dogs were led out of the testing room at the end of each trial, and after a rest period of five minutes they were reintroduced for the start of a new trial.

### 2.6. Data Collection and Analysis

Using Observer XT software (version 12.5, Noldus, Groeningen, The Netherlands) a continuous sampling technique was used to collect data about dogs’ visual orientation from the videos recorded during testing. Dogs’ visual orientation was coded as “left” if the dog was looking at the point-light display to the dog’s left, “right” if they were looking at the point-light display to the dog’s right, and “elsewhere” if the dog was looking anywhere else in the room. If at any time it was not possible to tell where a dog was looking by the frontal video, then head orientation (videos from above) could be used but this was rarely needed. Inter-observer reliability was assessed using data collected by a second observer for dogs’ visual orientation on 20% of videos and was revealed to be good (Pearson’s *r *= 0.85). Data collected about the dogs’ orientation were used to compute the total amount of time in which dogs looked at the stimuli as well the total amount of time the dogs looked at either stimulus. For the analysis, only data for dogs who looked at the display for a minimum of 5 s were included.

A generalised estimating equation (GEE) model was used to assess the influence of various physical characteristics of point-light displays on the amount of looking time dogs directed towards motion displays. In building the model, being scrambled (yes/no) and/or inverted (yes/no) were included as fixed factors, as were their first-order interactions. The dog’s ID was included as a random factor, to account for the repeated sampling from each dog. Bonferroni-corrected post-hoc comparisons were performed when a significant effect was found for any of the factors included in the model.

As humans and dogs were never presented in the same display, the “species” factor was not included in the GEE model described above, and separate models were run on data collected from trials where dogs and where humans were presented. However, an additional analysis was performed on the total amount of attention paid by dogs to either stimulus to determine whether the presentation of dogs or humans had an overall effect in attracting dogs’ attention. To this aim, a GEE model was run on total attention as dependent variable, the species displayed as a two-level factor, including the dog’s ID as random factor.

All statistical analyses were conducted using SPSS (version 24, IBM, Armonk, NY, USA), with statistical significance level set at 0.05. 

## 3. Results

An average of 15 dogs in each trial across all trial types (min: 10; max: 19) looked at the stimuli for more than 5.0 s. These dogs directed a minimum of 5.0 s towards both point-light displays, and a maximum of 15.0 s, with a mean ± SD of 9.6 ± 2.9 s, with no significant difference between displays showing dogs or humans (Wald χ^2^ = 0.277, *p* = 0.599). Of this looking time, dogs directed a minimum of 0.0 s towards each stimulus, and a maximum of 15.0 s (mean ± SD: 4.8 ± 4.2 s).

### Effect of Stimulus Properties on Looking Time

**Figure 3:**
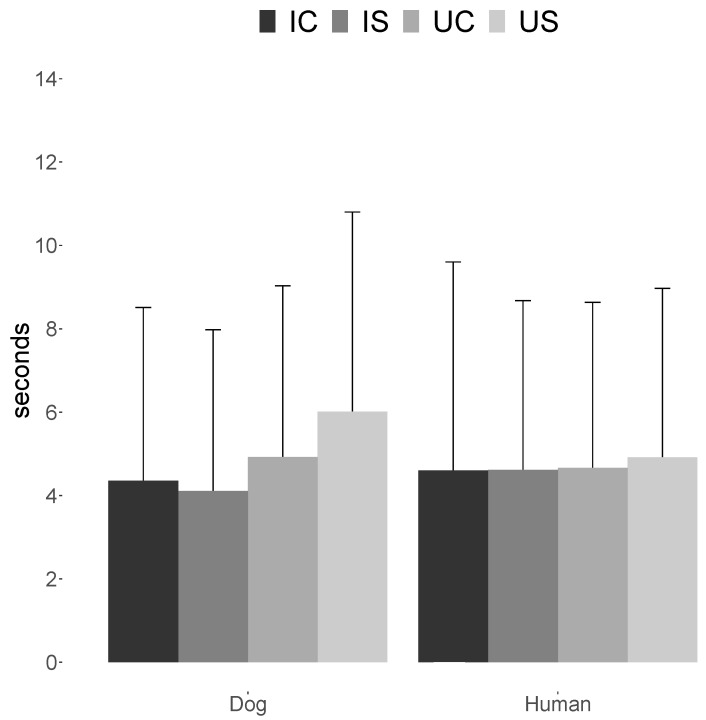
Mean ± SD amount of attention paid by dogs to upright-coherent (UC), upright-scrambled (US), inverted-coherent (IC) and inverted-scrambled (IS) light-point figures representing a walking dog or a walking human.

Results of the GEE indicating the effect of factors influencing dogs’ looking time towards human point-light displays are summarised in Table 2. With regards to human trials, there was no effect of inversion, scrambling or an interaction between the two at either time point. Results of the GEE indicating the effect of factors influencing dogs’ looking time towards dog point-light displays are summarised in Table 3. No effect was found on dogs’ looking time for scrambling and the interaction between scrambling and inversion at either time point. Conversely, a significant effect of inversion was found, with dogs preferentially looking at upright dog point-light displays (mean ± SE: 5.5 ± 0.4 s; 95% CI: (4.7, 6.2)) compared to inverted dog point-light displays (4.2 ± 0.3 s; 95% CI: (3.5, 4.8); mean difference ± SE: 1.2 ± 0.6 s; 95% CI: (0.1, 2.4)). Mean ± SD looking time towards dog and human point-light displays with different manipulations are presented in Figure 3.

## 4. Discussion

The current study investigated which features of biological motion the dogs’ directed more looking time towards, by presenting them with pairs of point-light displays of walking dogs or humans that contained aspects methodically manipulated (coherent/scrambled, upright/inverted). The results revealed that dogs directed significantly longer viewing times towards upright dog point-light displays, regardless of their global configuration. No bias in visual preference was observed when dogs were presented with any of the human point-light displays.

The finding that dogs significantly biased their looking time towards dog upright point-light displays corroborates with previous research which found that inverting point-light displays reduced the amount of looking time they attracted (e.g., chicks [20]; marmosets [23]) and impaired visual task performance (cats [11]; pigeons [14]). Traditionally, inversion was believed to impact an individual’s ability to process stimuli holistically, a renowned effect observed in face processing [29,30]. However, in the case of biological motion stimuli, the effect of inversion is still present, even when viewing scrambled point-light displays [26], when holistic perception would not be possible. More relevant to the perception of biological motion is that inversion alters the kinematic properties of the moving dots, which no longer move in accordance with the laws of gravity. Thus, a detrimental effect of inversion on viewing times indicates that accordance with gravity is crucial to the detection of biological motion, as observed in chicks and humans [20,31]. This seems also to be the case for dogs in our experiment.

Nevertheless, accordance with gravity as such is not sufficient to explain why our dogs showed a bias in looking time for upright dog point-light displays, but not for upright human ones. A first explanation could be focused on the movement of specific dots. Particularly, human and dog stimuli differed in the amount of limb motion they contained. Not only was this due to the fact that humans are bipeds, but even more feet motion was present in dogs’ point-light displays since two joint-dots were marked on every dog limb (ankle and metatarsophalangeal or metacarpophalangeal joints), whilst only one joint was marked on every human leg (ankle). Previous evidence showed that some point-lights provided more movement information than others, implying that there was something “special” about the motion of these dots. For example, Mather and co-authors [32] found that people selected the direction with an accuracy rate of 90% if the shoulder and hip dots, or elbow and knee dots were removed, but performed at near-chance levels if the wrist and ankle dots were removed. It could be argued that the arm movement of the human point-light display contains all the same biological movement that is contained in the legs, rising and falling under the influence of gravity. However, the kinematics of arms and limbs/legs movements are quite different. In accordance with this, Troje and Westhoff [26] also found that local feet motion was crucial for human participants to extract directional information from point-light displays, and based on this they suggested that the local motion contained in animals’ feet was used as part of an evolutionary system for detecting animals within their visual environment. This idea is supported by Chang and Troje [33] who claim that it is the vertical acceleration pattern which feet motion contains that is essential to allow the visual system to identity an animal. Also, an electroencephalographic (EEG) study by Wang and co-authors [34] found that humans automatically responded to the characteristics of the local biological motion, but not of the global configuration.

A further aspect that needs to be considered when discussing the lack of effects of scrambling, is ambient luminance. An earlier study showed that humans have difficulty in discriminating scrambled from unscrambled biological motion figures at very low light levels [35]. The authors argued that such conditions only affect the perception of local motion to a limited extent but make it more difficult to assemble local signals into a global percept. Whether this is true, and at what light intensities this occurs, is a matter of debate. For instance, Burton and collaborators [36] report an impairment in the perception of (global) biological motion only in the scotopic range (e.g., when only rod photoceptors are active). Conversely, Billino and collaborators [37] report the greatest impairment in the mesopic range, attributing it to the conflicting, simultaneous activation of the rod and cone systems. Our experiment was conducted at light levels slightly above the threshold between the mesopic and photopic range of humans [38], which would predict no detrimental effect on our dogs’ perceptual abilities. However, substantial differences exist between dogs’ and humans’ vision, including lower acuity at various luminance levels [39], and higher light sensitivity [40], which suggest that thresholds between the photopic, mesopic and scotopic range may also differ between the two species. In this sense, we cannot exclude that the relatively low ambient luminance contributed to the irrelevance of global configuration in driving dogs’ attention to biological motion stimuli.

Another hypothesis that could explain the difference in looking time for upright dog point-light displays, but not for upright human ones might be related to different patterns of neural activity which underlie visual processing of conspecific and heterospecific movements. Previous studies in humans [41,42] and monkeys [43,44,45,46] indicate that visual sensitivity to actions depends on the observer’s past motor experience with the action being observed. Using functional magnetic resonance imaging, Saygin and co-authors [47] found in humans that brain regions containing mirror-neurons are activated by viewing human point-light displays. Similar findings were also revealed during EEG studies with point-light displays showing different human actions [48] and emotions [49]. In line with these findings, Pinto and Shiffrar [50] found that people demonstrated greater visual sensitivity to coherent human motion than coherent horse motion perception in point-light displays. Thus far, mirror neurons have not been explicitly identified in dogs, but their presence can be assumed on the basis of dogs’ performance during experiments investigating emotional contagion and mimicry (e.g., [51]). It is then possible that human motion is not able to attract attention in dogs because it does not activate their mirror neurons since they cannot physically preform the action themselves.

Our results support the hypothesis that the characteristic motion of the limbs of an animal in locomotion are crucial for biological motion detection [26], although detection in dogs may be selectively tuned to other quadrupeds. Whilst the evolutionary usefulness of such capacity is clear, allowing dogs to quickly identify other animals within their environment and act on that information accordingly, it also suggests that neither history of domestication, nor adult pet dogs’ extensive exposure to humans, enhanced the salience of human bipedal motion in the same way. However, it remains unclear whether dogs recognised point-light displays as representing other dogs or at least a generic quadrupedal organism, or only perceived the characteristics of the local motion contained within the display. A study by Vallortigara and co-authors [19] suggests that chicks are not able to infer species information from movement, since they spontaneously approached point-light displays of predators as well as conspecifics. Further support for the idea that animals do not recognise the identity of point-light displays was provided by research in human infants [25] and chicks [20] which revealed no looking time bias towards upright coherent or upright scrambled/random point-light displays. The lack of any effect of scrambling in our experiment suggests that dogs may also not be able to recognise dogs in these types of stimuli.

At first glance, our results stand at odds with some previous findings. For instance, Kovacs and co-authors [16] suggested that dogs’ preference for unmasked human biological motion was reduced by the administration of oxytocin; however, no main effect for biological motion preference was found in the placebo group dogs which falls in line with what we observed during human point-light trials. Ishikawa and co-authors [17] reported an overall looking time bias towards upright humans compared to inverted point-light displays, which clashes with the lack of inversion effect for humans in our study. However, this finding was observed when the human point-light displays were presented in the frontal orientation which the current experiment did not include. When constrained to dog point-light displays, Ishikawa and co-authors found no significant preferences for lateral upright compared to inverted dog point-light displays. A possible explanation for the lack of any preference found in their work could be the difference in the procedure. Unlike Ishikawa et al.’s experiment where the point-light displays were presented for 5 s, the current study measured dogs’ looking time at 15 s time points allowing to capture more sustained looking time biases.

## 5. Conclusions

A systematic investigation of dogs’ looking time allocation towards different features of point-light displays revealed that dogs did not show any bias towards features of human point-light displays, but preferentially viewed upright biological motion displays originated from walking dogs, compared to inverted ones and regardless of the global configuration. In line with previous research in animals, the finding champions the importance of limb motion in accordance with the laws of gravity for the detection of moving biological entities by dogs.

In spite of some discrepancies between the present findings and those of other studies in dogs, ours and previous research do converge on one remarkable aspect: that the extent of dog’s attentional biases towards biological motion displays is rather limited. Considering that visual preference to biological motion seems well conserved across taxa, it seems unlikely that such weak bias merely reflects a scarce sensitivity of the species towards biological motion. Rather, other factors may have contributed to drive dogs’ attention towards visual stimuli; for instance, both the novelty of a specific stimulus, as well as the familiarity of the subject with a certain category of stimuli, can interact in determining dogs’ preferential looking at certain visual displays [52]. Thus, in ours, as well as in past research, these factors may have been competing to some extent with the attractiveness of figures depicting biological motion.

## Figures and Tables

**Table 1:** Combinations of stimuli presented in the six different trial types. Trials featuring these stimuli were created for both dog and human point-light displays. UC = upright coherent, IC = inverted coherent, US = upright scrambled, IS = inverted coherent.

Trial Type	Stimulus 1	Stimulus 2
1	UC	IC
2	UC	US
3	US	IC
4	US	IS
5	IS	UC
6	IS	IC

**Table 2:** Results of the Generalized Estimation Equation model on looking time to each stimulus during human trials. df = degrees of freedom.

Factor	Wald χ*^2^*	df	*p*-Value
Inverted human	0.058	1	0.810
Scrambled human	0.400	1	0.841
Inverted × Scrambled human	0.023	1	0.880

**Table 3:** Results of the Generalized Estimation Equation model on looking time to each stimulus during dog trials. df = degrees of freedom.

Factor	Wald χ*^2^*	df	*p-*Value
Inverted dog	4.198	1	0.040
Scrambled dog	0.347	1	0.556
Inverted × Scrambled dog	0.856	1	0.355
